# Information-Centric Network-Based Vehicular Communications: Overview and Research Opportunities

**DOI:** 10.3390/s18113957

**Published:** 2018-11-15

**Authors:** Ikram Ud Din, Byung-Seo Kim, Suhaidi Hassan, Mohsen Guizani, Mohammed Atiquzzaman, Joel J. P. C. Rodrigues

**Affiliations:** 1 Department of Information Technology, The University of Haripur, Haripur 22620, Khyber Pakhtunkhwa, Pakistan; ikramuddin205@yahoo.com; 2 Department of Software and Communications Engineering, Hongik University, Sejong 30016, Korea; 3 InterNetWorks Research Laboratory, School of Computing, Universiti Utara Malaysia, Sintok, Kedah 06010, Malaysia; suhaidi@uum.edu.my; 4 College of Engineering, Qatar University, Doha, 2713 Qatar; mguizani@ieee.org; 5 School of Computer Science, University of Oklahoma, Norman, OK 73019-0390, USA; atiq@ou.edu; 6 National Institute of Telecommunications (Inatel), Santa Rita do Sapucaí 37540-000, Brazil; joeljr@ieee.org; 7 Instituto de Telecomunicações, 1049-001 Lisbon, Portugal; 8 University of Fortaleza (UNIFOR), Fortaleza 60811-905, Brazil

**Keywords:** information-centric network, VANET, SDN, cloud, edge, 5G

## Abstract

Information Centric Network (ICN) is expected to be the favorable deployable future Internet paradigm. ICN intends to replace the current IP-based model with the name-based content-centric model, as it aims at providing better security, scalability, and content distribution. However, it is a challenging task to conceive how ICN can be linked with the other most emerging paradigm, i.e., Vehicular Ad hoc Network (VANET). In this article, we present an overview of the ICN-based VANET approach in line with its contributions and research challenges.In addition, the connectivity issues of vehicular ICN model is presented with some other emerging paradigms, such as Software Defined Network (SDN), Cloud, and Edge computing. Moreover, some ICN-based VANET research opportunities, in terms of security, mobility, routing, naming, caching, and fifth generation (5G) communications, are also covered at the end of the paper.

## 1. Introduction

**Figure 1:**
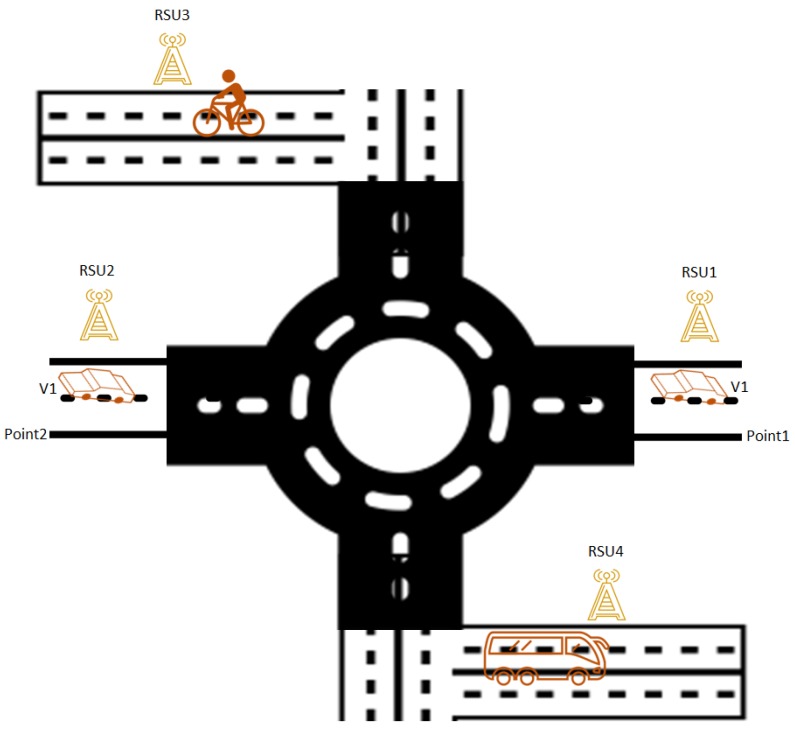
Vehicular internet.

In Information-Centric Networking (ICN), content is retrieved based on its name rather than IP address [1]. As the Internet shifts from IP-based communication to a content name-based approach, this paradigm will face critical challenges in some emerging environments, such as Vehicular Ad hoc Networks (VANETs). That is, vehicles run at a very fast speed, thus, retrieving contents from a local caching node is quite challenging. A few more issues are associated with caching in the ICN-based VANET environment, for example, mobility, security, routing, naming, and caching. To explore this point more, let us consider the scenario in Figure 1. A vehicle V1 retrieves content, which is supposed to be already cached in RSU1. After moving from point1 to point2, V1 is disconnected with RSU1 and establishes a link with RSU2. However, RSU2 does not have the desired content of V1. Therefore, this mobility issue is addressable before the ICN deployment in the VANET environment.

Similarly, routing V1’s desired content from RSU1 to RSU2 is also challenging, as it is not known that V1 is now connected to RSU2 so that the content should be forwarded there for V1’s satisfaction. Moreover, spoofing and eavesdropping are the basic challenges in every kind of network, thus, “*how these issues should be resolved when deploying ICN for VANETs?”* is a thinkable question.

The rest of the paper is structured as follows: Section 2 presents the integration of ICN and VANET; Section 3 elaborates the issues faced when ICN is integrated with VANET; Section 4 reports research challenges when ICN-based VANET is to be deployed with the other three architectures, i.e., Software Defined Network (SDN), Cloud, and Edge; Section 5 signifies the challenges that may be faced during the ICN-based VANET deployment; Section 6 discusses the lessons learned; Section 7 concludes the paper.

## 2. ICN-Based VANET

The research community and academia world explored and successfully deployed VANET in the last two decades. Currently, it is gaining more popularity due to rapid increase in the number of vehicles worldwide. VANET emerges in our daily life through conferring vehicles and their related objects, for example, road side units (RSUs), with communication resources that lead to vehicle-to-vehicle (V2V), vehicle-to-infrastructure (V2I), infrastructure-to-vehicle (I2V), and more generically, vehicle-to-everything (V2X) communications. Sensors in vehicles allow the transmission of collected information such as live video streaming between two vehicles, V2X application servers, RSUs, and cellphones of pedestrians [2]. Vehicles may extend the insight of their network outside and can have more general view of the local settings. Remote driving permits an application concerning V2X to activate a remote vehicle located in a hazardous zone or for people who are unable to drive vehicles. For a scenario where roads are predictable such as public transportation, then driving on the basis of cloud computing may be used. For this kind of scenario, such a platform may be considered where the entrance is based on cloud services [2].

VANET related applications have several advantages, for instance, it reduces accidents and harms identified with individuals and vehicles [3]. In addition, it saves individuals time by providing traffic related data such as a busy and congested road [4].

ICN is an appealing applicant solution for vehicular communications due to its numerous benefits. First, it fits fine to the quality of usual VANET usages, such as route reports and accident messages [5]. These usages are probable to benefit from in-network content caching and strategies. Second, data caching is mainly favorable to speed up content retrieval via caching in different nodes [6]. In vehicles, caching may typically be deployed at fairly low cost, as the energy demands of ICN nodes are likely to be a small portion of the overall energy use of vehicles, hence agreeing on high-level computation, uninterrupted data processing, and enough caching space in vehicles. In addition, ICN mainly endorses asynchronous information sharing among end users. [5,7].

Beside several advantages, VANET also faces numerous challenges, for example, vehicles’ mobility and network disruptions, i.e., if two vehicles are connected in a grid and after some distance they change their route, then it is a challenging task that how they can communicate with each other. To overcome these kinds of issues, a new clean-slate model is required so that the user quality of experience is achieved. ICN is believed to be the most suitable paradigm for smooth data transmission without extra retrieval delay in VANET environments.

## 3. ICN-Based VANET Issues

The existing papers, such as [8,9,10] address the ICN features corresponding to VANET approach, which motivate this article. However, these papers present VANET issues in part or whole in terms of security, routing, mobility, and scalability. In this paper, we investigate the ICN-based VANET challenges with respect to security, routing, mobility, naming, and caching, which are serious issues and must be resolved when ICN is deployed in the VANET environment. The mentioned modules have received a tremendous interest from the VANET research community that works towards the ICN-based VANET deployment.

Security, among other features, is the most vital feature of a wireless sensor network, which can be resolved up to a maximum extent. However, there are still some scenarios in which ICN may also face problems. These issues can be categorized into several modes, such as denial of service (DOS) attacks. These attacks may be further divided into numerous types, for example, attacks on (a) authentication, (b) availability, and (c) confidentiality. Authentication is further divided into Sybil and impersonation. In the prior one the attacker exercises various identities at a time, whereas in the latter one the attacker demonstrates himself as the genuine user. Availability is the most vigorous element in the VANET environment, therefore, attackers exercise attacks on this critical aspect. The main aim of attackers in this module is to confirm customers’ lives. This element has been presented in the literature rigorously, and interested readers are referred to [10] for in-depth comprehensions. In the third module, confidentiality, the information is merely accessed by the authorized group. In other words, the information must be hidden from unauthorized users. Confidentiality is prone to attacks due to exchanged information in the public network. However, attacks on the confidentiality may be avoided up to a maximum level in ICN as content requests are based on names rather than IP addresses.

ICN routing mechanisms are categorized into two classes, i.e., name-based routing and name resolution. In name-based routing, a content request is forwarded on the basis of content name where its related information is stored on the publisher-subscriber path and therefore the content itself is delivered on the reverse path to the subscriber [11]. On the other hand, name resolution can be achieved in two steps. In the first step, the content is matched with a single IP or group of IP addresses. In the second step, a shortest path in the network is followed using any protocol such as Open Shortest Path First (OSFP), and subscribers’ requests are forwarded to the content publisher [12].

The existing Internet was designed for fixed devices, where a node’s IP should be in the subnet of the network to which it is attached. Nevertheless, the number of non-fixed nodes shows a persistent growth where wireless traffic with 27 billion devices will report for more than 63% of the total IP traffic by 2021 [13]. Mobile devices can easily switch networks, change their IP addresses, and therefore present novel transmission means on the basis of opportunistic and intermittent connectivity [14]. Nonetheless, such kinds of approaches do not attain uninterrupted connectivity, which has become an essential need to a great extent.

ICN naming may be divided into two classes, i.e., hierarchical and flat naming. In the hierarchical approach, a name can be made of several hierarchical elements. Where an element may be a series of characters that is created by subscribers. Hierarchical names are easy to understand but they are non-persistent [12]. On the other hand, a flat name approach is more useful as compared to hierarchical one because a hash-table is used to identify the next hop in case of a content request [15]. The supremacy of flat names over hierarchical names is such that flat names can be subdivided and therefore parallel processing can be achieved.

ICN In-network caching can be achieved using three principles, i.e., democratic, uniform, and pervasive [16]. In the democratic principle, all network nodes have equal rights to publish contents if they have cached them already. In the uniform principle, a routing protocol may be used for all contents or all network nodes, if required. Pervasive principle demands that a cached content should be available/provided to all nodes in the network.

## 4. An Overview of Research Challenges

**Figure 2:**
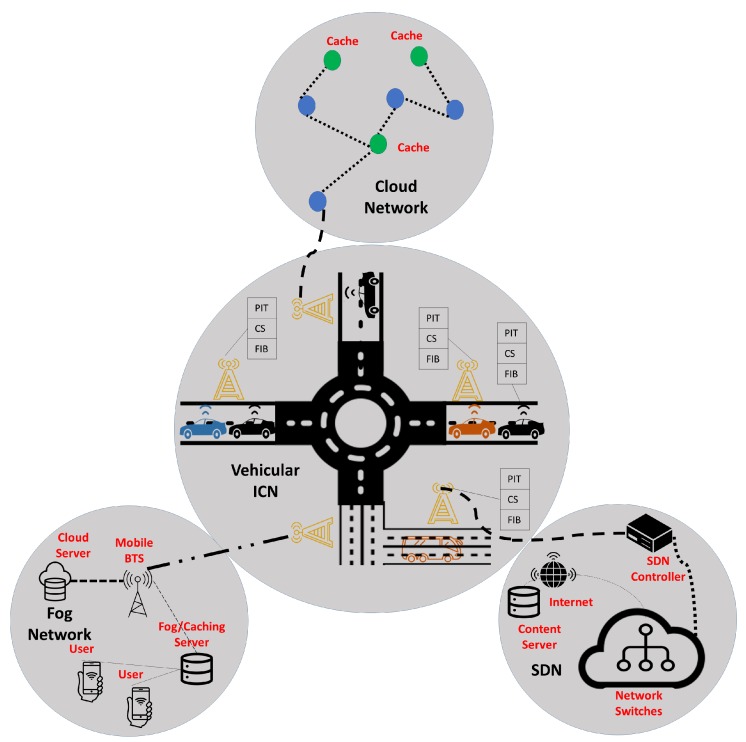
Integration of other technologies with Vehicular ICN.

**Figure 3:**
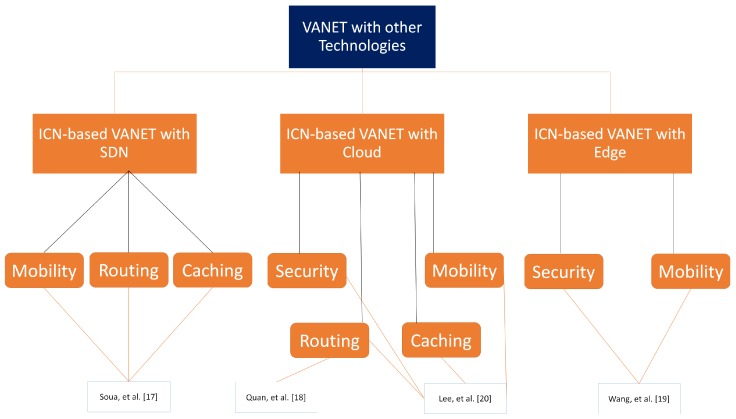
A taxonomy of vehicular network with other emerging technologies based on literature survey.

In this section, we provide the imperative ICN-based VANET challenges, as presented in Figure 2, that need to be addressed and resolved before the ICN deployment. The main goal is to grasp the attention of the ICN, SDN, Edge, and VANET research communities for merging these attractive models at one platform. The taxonomy of these models is presented in Figure 3, which is based on the papers [17,18,19,20].

In ICN, contents are forwarded hop-by-hop by in-network nodes, with each node holding three data structures, i.e., Pending Interest Table (PIT) that tracks records of the interfaces through which content requests arrive, Forwarding Information Base (FIB) that matches content names to the output interfaces, and Content Store (CS) which is used to cache contents locally [14].

### 4.1. SDN-Based Vehicular ICN

SDN carries a new idea to the Cloud [21] by presenting resource separation where all resources are assembled and supervised by software. Unlike legacy physical server model, SDN takes into account resource separation and allows for hardware resources as discrete and flexible elements. With the deployment of SDN, the concept of client-server system is exterminated, but only information of various hardware is coded and processed. The administrator of a Cloud architecture then deploys a particular client-server environment, for example, logical clients and servers, which brings a high level of flexibility to the Cloud environment. However, this phenomenon needs essential physical resources so that to connect various devices through a high-speed communications channel to deal with the physical disaggregation of resources [21].

Moreover, SDN is an evolving idea that isolates data plane from control plane, where the controller collects information from network nodes and provides an abstract view of the network. In addition, all SDN applications can access the SDN controller, where the applications deploy various network services [20]. In the context of ICN-based VANET, SDN provides scalability, manageability, and a universal view of the network. Among different ICN modules, in-network caching is one of the most popular approach [22], which improves the content availability ratio and decreases the content retrieval delay. SDN is the most suitable choice for the cache improvement as it helps in distinguishing diverse kinds of cached contents. However, this linkage of ICN and SDN in the VANET environment is a challenging task due to vehicles’ mobility.

An SDN-based VANET scheme is proposed in [23], where different wireless nodes are considered as SDN switches, which share the network bandwidth in a centralized control plane. This technique seems suitable for the SDN-based VANET, but it does not consider the most promising paradigm, i.e., ICN. Thus, an intelligent and flexible method is required for the ICN-based VANET communication paradigm to share network resources with smooth content mobility and caching.

### 4.2. Cloud-Based Vehicular ICN

Unlike data processing/execution in a local area network (LAN), Cloud computing is a concept where computation depends on difference resources that are shared in a wide area network (WAN). Usually, the architecture of Cloud computing is based on across-the-board data centers connected together wherein users access their desired resources through the Internet [24]. At present, various companies such as Google, Microsoft, and Amazon exploit Cloud data centers [25] for caching a huge amount of records and hold prevalent service applications [26]. Because of the hardware and/or software constraints or reformation of security issues, it is indispensable to use data centers for storing records [25,26]. However, service provision in this situation becomes a crucial problem.

Due to exponential growth in Internet traffic, use of vehicles as cloud nodes is an accepting idea. This is possible because several vehicles are equipped with caching and sensing sensors for providing safety in the driving as well as infotainment for passengers. Using the cloud as an *infotainment-only* feature is easy in the current IP-based Internet paradigm. However, linking this feature with the ICN model is a challenging task due to vehicles’ mobility. Using the concept of naming in ICN, seamless mobility can be supported without executing difficult network administrations needed in the IP-based networks, when topological or physical locations of mobile nodes change [27]. In the last few years, various strategies have been proposed for addressing this challenge from the viewpoints of publisher and subscriber mobility. The proposals for enabling subscriber’s mobility generally incorporate proactive caching [28] and prompt recovery of request/reply [29].

Basically, ICN is a publish-subscribe networking model, wherein subscribers are mainly interested in actual contents rather than their locations [30], its primary focus is on content retrieval to allow subscribers to attain the requested information. Thus, it is a prospective model to resolve several ubiquitous challenges face in the IP-based infrastructure, for example, mobility and security among others [27].

Mobile cloud services can be integrated with the ICN-based VANET to provide access to the stored information. However, *due to the nature of ICN accessing method which is based on names*, a feasible connectivity approach is required for the smooth transition. Nevertheless, this facility raises several new challenges in the network model, which are discussed in Section 5.

### 4.3. Edge Computing-Based Vehicular ICN

Edge computing [31] is deployed to bring computing resources near data subscribers. The architecture of Edge computing is decentralized that uses network nodes to jointly execute a considerable amount of processing, data caching, and controlling [32,33]. With the help of Edge nodes and network connections, processing overhead is largely decreased and bandwidth restrictions are surmounted for consolidated services [19]. The attraction of Edge computing increases with the provision of on-demand services and availability of resources near consumer devices, which result in low response time and greater consumer satisfaction [34].

Data sharing in vehicular network has been a growing concern for the last few years. Three types of data sharing are deemed the most prominent practices in vehicular network, i.e., sharing warning messages about an accident, reminder for the prevention of vehicles crash, and notice about the road congestion [35]. Besides, infotainment content availability for passengers has also attracted the attention of vehicular research forums. In this regard, Edge computing, which brings the storage capacity and computational processes near the customers for content retrieval with minimum delays, is integrated with vehicular networks [36]. However, the integration of ICN with vehicular Edge computing is a challenging issue due to consistent mobility of vehicles. That is, if content, which is stored in an Edge node (a vehicle), is accessed by a passenger in a moving car, that can be accessed somehow easily in the IP-based Internet model. However, relating it with the ICN, which believes in names rather than IPs, is quite complicated job. This can be resolved by storing an accessed content at several RSUs thereby ignoring the redundancy factor. Yet, according to the Cisco Visual Networking Index (VNI) [13], mobile traffic by 2021, with 5.5 billion users, will reach seven times more than the current traffic, which introduces further complication for vehicular ICN. In other words, the storage of *RSUs* would exhaust in a moment, which requires replacement of the *already-stored* contents. In this scenario, the accessing sensor node (vehicle) will not find the previously cached content. This scenario motivates researchers to design such an intelligent and sophisticated mechanism that paves the way to integrate Edge and ICN with vehicular networks.

## 5. ICN-Based VANET Research Opportunities

**Figure 4:**
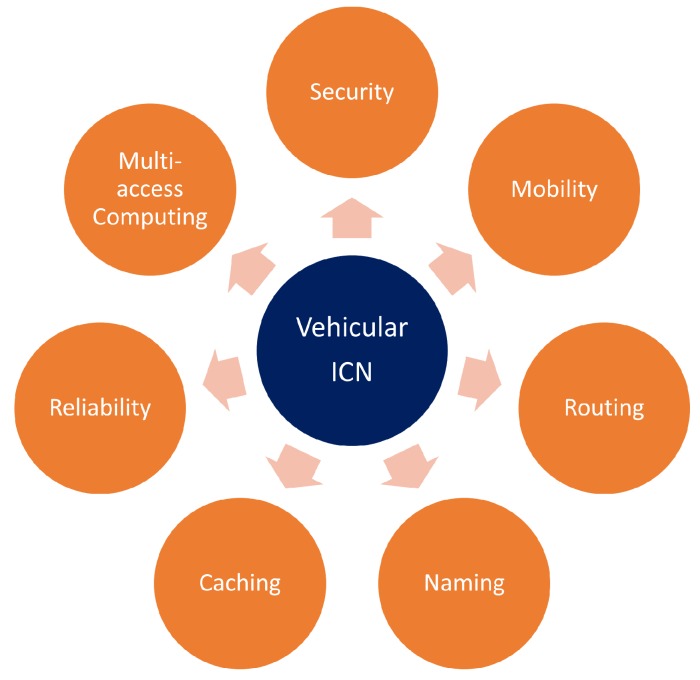
An example of research challenges faced by Vehicular ICN.

Generally, VANET communications are achieved on the basis of two key techniques, i.e., cellular networking and dedicated short range communications (DSRC) [37]. Through these two technologies, a vehicle obtains information from its own sensors and provides it to other vehicles or RSUs [38]. Currently, moving computing, management, and content caching in the Edge, ICN, and Cloud is the growing trend. This computation however brings a lot of new system requirements, which are presented in Figure 4 and discussed in the following subsections.

### 5.1. ICN-Based VANET Security

Numerous Internet security issues are mainly caused by data disruption at the application layer, which is primarily happened because of the IP—a best effort delivery but unreliable protocol. On the other hand, as ICN is a content-driven paradigm, content is published when it is requested. This approach curtails the transmission of unsolicited content objects and thereby automatically provides content security, which is achieved by using content names. In addition, ICN also affords malicious content filtering through in-network security policies [39]. Furthermore, ICN adds a point of indirection among the subscribers of requested contents, i.e., the content requests are assessed by in-network nodes before reaching the publisher [11]. As discussed above, coupling of ICN is possible with the Internet of vehicles, and this functionality offers opportunities for researchers to design an adaptable mechanism for their association.

### 5.2. ICN-Based VANET Mobility

Mobility is a critical challenge to the ICN deployment corresponding to all emerging technologies [40]. In ICN, when subscribers change their location, their connectivity is changed from one node to the other. However, as no IP address is used to route contents, it is transparent, in contrast to IP, in which addresses are changed [41]. In the VANET environment, content objects must pass a centralized facilitator (vehicle having sensors) before reaching the actual subscriber. This is the most crucial module because contents in VANETs travel along a longer path rather than the best one. Mobility in ICN is achieved through a paradigm, known as publish-subscribe Internet model. In this approach, interested subscribers request particular contents by sending request messages without knowing the location of the content, and the publisher responds with the actual content [41].

This phenomenon guarantees secure content distribution because of the publisher and subscriber decoupling, and thereby catch the attention of the ICN and VANET communities for integrating these emerging models. However, providing mobility support in ICN-based VANETs requires smart and suitable techniques so that content should route to a particular destination without extra retrieving delay.

### 5.3. ICN-Based VANET Routing

ICN considers in-network caching– an approach that encompasses replicas of the accessed contents in the process of data routing [9]. An in-network node in ICN can satisfy subscribers’ content requests by looking for those particular sources where the contents are cached. Once content is delivered to the requested subscriber, a path between the content publisher and subscriber, and a specific interface from which the request was received, are recorded for the execution of future requests [42]. This path is recorded by looking at the network condition. The contents in ICN are accessed through names rather than IP addresses, which help in combining requests for a specific content and enable its provision to the corresponding subscribers through multicast forwarding mechanisms.

However, this forwarding in the VANET environment raises more challenges due to instant mobility of caching sensors (vehicles). Thus, the research community is encouraged to design forwarding mechanisms by considering mobility and caching modules for the swift transmission of content objects.

### 5.4. ICN-Based VANET Naming

Unlike the current Internet sending-receiving approach, contents in ICN are named, where names are either hierarchical or flat [43]. In ICN, the name of the content is decoupled from its location with the intention to supply it to the requesting subscriber. Hence, content retrieval follows a receiver-driven approach and therefore avoids control of subscribers over the content, as done in the IP-based architecture. In the ICN-based VANET, content may be easily discovered as compared to the IP-based VANET, because ICN does not need the original server (publisher) to be connected every time content is requested. Furthermore, content retrieval from different publishers, for instance, map from a common RSU, becomes easier through combining requests for contents with the same names. This process simplifies the process of data delivery for the incoming requests [9].

ICN naming brings significant assistance to vehicular communications by allowing forwarding vehicles to handle contents on the basis of application requirements. Named data transmission provides ICN-based VANET with robustness to connection interruptions and hereby characterizes vehicular Internet [44].

### 5.5. ICN-Based VANET Caching

ICN caching is divided into several parts, i.e., (a) off-path caching that requires extra storing devices, (b) on-path caching which is achieved opportunistically, (c) homogeneous caching where two caching nodes co-operate with each other, and (d) heterogeneous caching in which caching nodes do not co-operate with one another. For understanding the concept of these techniques, a recent survey [22] provides a detailed explanation of ICN caching strategies along with their contributions and limitations.

In vehicular networks, caching is achieved either through RSUs or even another sensing device such as vehicle. If an RSU is deemed as a caching node and a vehicle needs to access its cached data, then it can be acquired easily [38]. However, when a vehicle covers some distance and leaves the covering area of the RSU, then it is complicated to locate the real cache position. Similarly, if another vehicle is supposed to be a caching node, then after covering distance in the opposite direction from the accessing vehicle, it is more challenging to locate that caching vehicle. Moreover, cache update is also an essential mania for avoiding traffic congestions and accidents, which may cause due to outdated cached content. Therefore, a flawless updating cache strategy accompanied by a fine-grained forwarding mechanism is the basic requirement of the ICN-based VANET environment.

### 5.6. ICN-Based VANET Reliability

The deployment of ICN schemes in VANETs confronts the most vibrant and heterogeneous kind of problems, when considering the goals and requirements of such integration [5]. The difference in techniques (with respect to actuators, sensors, end-to-end diversity, and their functionalities, and in the collected and consumed data in such scenarios, would certainly lead to various concerns [5]. For example, vehicular nodes share similarities with the restrictions and prerequisites. One of the most vital problems is the use of technologies that may deploy resourceful connectivity in an unreliable network, i.e., VANET.

Nevertheless, one of the most significant points in vehicular communications is how different semantics of the shared and stored contents would affect content distributions. In reality, this is a challenging issue which is not covered in the ICN scope [45]. Considering the facility of in-network nodes to store forwarded contents to increase upcoming requests, an issue appears concerning the ICN framework that whether it should participate in any type of process that enables the association or the analysis of various suppliers of information [5].

In VANETs, the traffic on wireless medium that results from periodic packet exchange needs to be wisely controlled so as to avoid decline in the quality of safety-related data at the time of receiving [46]. For this reason, various strategies have been proposed in the literature such as D-FPAV [47], ATC [48], and PULSAR [49], which regulate traffic congestion with a stringent fairness measure that needs to be achieved for security purposes as well as emergency messages. However, the exchange of control messages in vehicular ICN is yet-to-resolve issue and needs careful considerations for designing adaptive strategies to achieve fair and reliable transmissions.

### 5.7. ICN-Based 5G-Enabled VANET

The 5G architecture, proposed by 3GPP, provides manageability to familiarize new control plane and user plane functions in the perspective of network slicing, which offers better elasticity to control various applications as well as devices. Thus, ICN would benefit 5G from the viewpoint of multi-access edge computing (MEC) in terms of edge computing, edge caching, and session mobility [50]. In addition, mobile nodes are positioned at the network edge that support various delay sensitive applications, e.g., virtual and augmented reality (VR/AR), and driving of autonomous vehicles. [51].

This drift is useful in low-latency and high-bandwidth applications, e.g., VR/AR, and non-real-time applications, for instance, IoT communications and video-on-demand (VoD) [50].

Furthermore, the caching feature of ICN assists both real-time and non-real-time applications every time there are temporal or spatial associations among data objects retrieved by edge subscribers [50]. This argument can be strengthened by the study conducted in [52], where it is argued that vehicular named networking encodes the data of geolocation into names. This is important as all request messages are sent to geolocations where contents are published. This is utterly feasible that a request message hits a vehicle with the desired content prior to reach its location.

Moreover, the existing deployments of mobile communications hold session mobility through centralized techniques for routing, which face severe problems at the time of replication of service demands. Different from this phenomenon, name resolutions and application-restricted identifiers divide the notion deemed for ICN is exposed to grip node mobility in an effective way [53]. However, this module is quite challenging in such environments where rapid mobility is involved, for example, vehicular communications [54]. Thus, substantial determination is demanded in this area from the ICN community working on vehicular communications.

A summary of the existing ICN-based vehicular communication proposals is presented in Table 1.

## 6. Lessons Learned

The ICN-based vehicular network is a part of human-centric communications that use private and public-scale participating information gathering. This type of data network is driven by the production of high-performance nodes. Currently, these nodes contain billions of smartphones and now growing into smart meters, GPS in vehicles, and activity- supervising sportswear accessible to subscribers [57]. Drivers and vehicles can use the information, such as weather, road, traffic, and health conditions, on the road. Vehicular ICN has several applications [57], for example, vehicles can distribute the collected data via the network though pull-based or push-based mechanisms, they can interact with neighboring vehicles in a wireless fashion so as to send or receive road or traffic information.

ICN is a name-based communications paradigm that allows caching contents at local in-network nodes. With technological advancements, Internet is evolving in every field, which introduces the concept of Internet of Things (IoT). Things ranging from a small body-fit gadget to large vehicles. In addition, as the Internet shifts to name-based model (i.e., ICN) from the existing nature of IP-based communication, the combination of VANETs with the ICN may face various issues such as intermittent connectivity due to rapid mobility of vehicles. In this study, we investigated the ICN-based VANET challenges in terms of mobility, security, routing, naming, caching, and 5G communications.

Furthermore, due to several other communications enigmas such as security, DoS attacks, and exponentially increasing number of Internet users, the integration of ICN-based VANETs with other architectures, i.e., SDN, Cloud, and Edge, is the need of the day. We presented the idea of these architectures with the ICN-based VANET in line with their glitches that may probably be faced when deploy together.

## 7. Conclusions

In this paper, we addressed a promising paradigm, Vehicular Ad hoc Network (VANET), based on Information-Centric Network (ICN), with its potential deployment in three other emerging models, i.e., Software Defined Network (SDN), Cloud, and Edge computing. We explored the applications of VANET in the mentioned three architectures corresponding to ICN that face numerous problems in the IP-based Internet model. VANET-based ICN influences contents in the stated models in a perfect way among VANET nodes (e.g., RSUs and vehicles). We presented the ICN-based VANET concept along with its challenges in terms of security, mobility, routing, naming, caching, 5G communications. However, the practicality of ICN-based VANET with SDN, Cloud, and Edge imposes more elaboration and requires detailed analysis as these models are not fully developed.

References

## Figures and Tables

**Table 1:** Summary of ICN-based Vehicular Communications Research [9].

ICN Module	Reference	Contributions	Challenge
**Security**	Wang et al. [55]	Integrity checks on the basis of	Analyzing security threats
		public key infrastructure (PKI)		
**Mobility**	Tyson et al. [40]	Native mobility support	Two key differences of vehicular ICN
**Routing**	Amadeo et al. [56]	Collision avoidance	Techniques to avoid the explosion of ICN data structures
	Amadeo et al. [29]	Selective flooding scheme	Selection of dynamic outgoing interfaces
**Naming**	Yan et al. [57]	Hierarchical naming schemes	Agreement on common naming
	Quan et al. [58]	Flat naming scheme	Agreement on common naming
**Caching**	Yu et al. [59]	Caching unsolicited contents	Smart scope-based caching strategies
